# Transcriptome Sequencing to Detect the Potential Role of Long Noncoding RNAs in Salt-Sensitive Hypertensive Rats

**DOI:** 10.1155/2019/2816959

**Published:** 2019-12-06

**Authors:** Hao Wu, Sibo Zhu, Rui Yuan, Yang Yi, Hanqing Wang, Bo Gu, Timing Zhen, Kaichen Xing, Jun Ma

**Affiliations:** ^1^Department of Nephrology, Jing'an District Central Hospital of Shanghai Affiliated to Fudan University, No. 259, Xikang Road, Jing'an District, Shanghai 200000, China; ^2^School of Life Sciences, Fudan University, Shanghai 200438, China; ^3^Shanghai Cinoasia Institute, Shanghai 200438, China

## Abstract

**Backgrounds:**

Long noncoding RNAs (lncRNAs) play an important role in various biological processes. However, their functions in salt-sensitive hypertension are largely unknown. In this study, the lncRNA-seq technique was employed to compare the expression profiles of lncRNAs and mRNAs in salt-sensitive hypertensive rats.

**Methods:**

Blood pressure, serum sodium, and urinary creatinine were texted in salt-sensitive and salt-insensitive rats fed with different salt concentrations. High-throughput sequencing was used to detect the expression of lncRNAs and mRNA in the renal medulla of the two groups.

**Results:**

Blood pressure and urinary sodium/creatinine of high-salt diets of the sensitive group were significantly higher than that in the control group. Serum sodium has no significant difference between the two groups in high-salt diets. NONRATG007131.2 and NONRATG012674.2 were the most different lncRNAs in the high salt-sensitive group. Correlation analysis reveals that Matn1, Serpinb12, Anxa8, and Hspa5 may play an important role in salt-sensitive hypertension.

**Conclusion:**

This study analyzed the difference in lncRNA and mRNA between salt-sensitive and salt-insensitive rats with different salt diets by high-throughput sequencing. Salt sensitivity and salt concentration were two key factors for the induction of hypertension. We found some potential genes that play an important role in salt-sensitive hypertension.

## 1. Introduction

Hypertension is defined as a complex disease determined by both environmental and genetic factors. It has become a significant public health problem because of the high prevalence and increased risk of cardiovascular and renal diseases [1]. Salt intake plays a critical role among environmental factors; a lot of studies from animals and clinic trials reveal a positive relation between salt intake and hypertension [2–4]. However, not all individuals (whether normotensive or hypertensive) have the same sensitivity to salt intake [5]. Salt sensitivity refers to the different blood pressure responses that occur among individuals for salt stress or salt restriction, and it was first proposed by Lufl and Kawasaki in the late 1970s [6].

Since Kawasaki and Lufl recognized the heterogeneity of the blood pressure response to salt intake and then elaborated on the concept of salt sensitivity, a considerable amount of research studies have been accumulated by scholars. Various mechanisms relating to salt-sensitive hypertension have been proposed in recent decades [7] In traditional views, the mechanism of salt-sensitive hypertension is mainly associated with the renin-angiotensin-aldosterone system [8], sympathetic nervous system [9], renal sodium transport [10], and endothelial dysfunction [11]. A new research study shows high-salt dietary can result in hypertension by altering the intestinal microbiota in humans and mice [12].

Although these mechanisms contribute to evidence of blood pressure homeostasis and candidate genes in hypertension, they still fail to explain the role of most genes in changing blood pressure [13]. So exploring the role of genes in hypertension and controlling blood pressure with genetic methods is a crucial breakthrough we have sought in recent years. Long noncoding RNAs (lncRNAs) refer to the nonprotein-coding transcripts that are longer than 200 nucleotides, and they are considered to be nonbiologically functional by-products of the transcription process before. In recent years, they have been demonstrated to participate in posttranscriptional regulation, cell growth, differentiation, and proliferation of human genes [14, 15]. It has distinct tissue specificity and development stage specificity compared to protein-coding genes. The tissue specificity and developmental stage specificity are more apparent in long noncoding RNAs when compared to mRNAs. It also can regulate the expression and modification of protein-coding genes from multiple perspectives, such as epigenetic regulation, transcriptional regulation, and posttranscriptional regulation [16, 17]. Therefore, the study of long noncoding RNA is likely to be a breakthrough in salt-sensitive hypertension.

Some scholars also confirmed the importance of lncRNAs in salt-sensitive hypertension, and they have reported on the relationship between lncRNAs, salt-sensitive hypertension, and kidney [18–20]. Among these studies, two studies are related to the screening of lncRNAs in salt-sensitive hypertension. Gopalakrishnan et al. explored the differential expression of lncRNAs between Dahl-ss rats and SS-13BN rats [20]. Wang et al. studied the differential expression of lncRNAs in Dahl-ss rats, SS-13BN rats, and spontaneously hypertensive rats [21]. Although they are both related to lncRNAs screening, there are several shortages in their researches. First, Hiseq2000 was used in the process of their RNA sequencing, and we rarely use it now due to its inaccuracy. Second, Wang's experimental rats may have a shorter feeding period (7 days), and salt concentration is not very high (4%). Finally, their studies only performed RNA sequencing and failed to collect and analyze some observed indicators (such as blood pressure, blood/urine sodium, and blood/urine creatinine).

In the present study, a higher salt concentration diet (8%) was used to establish hypertensive models in salt-sensitive and salt-insensitive rats; the grouping of this study is more detailed (high-salt and low-salt diets were fed in sensitive and control groups, respectively) in order to facilitate accurate comparison; Hiseq3000 was applied in lncRNA sequencing, the observation period (2 months) of this study is more extended than any reported studies, and more indicators were collected to confirm results. By sequencing the medulla lncRNAs in rats, we found differentially expressed lncRNAs in two groups of rats and its associated mRNAs and related pathways. This study provides new therapeutic targets for elucidating the mechanisms of salt-sensitive hypertension-induced renal injury.

## 2. Materials and Methods

### 2.1. Grouping of Experimental Animals

**Figure 1:**
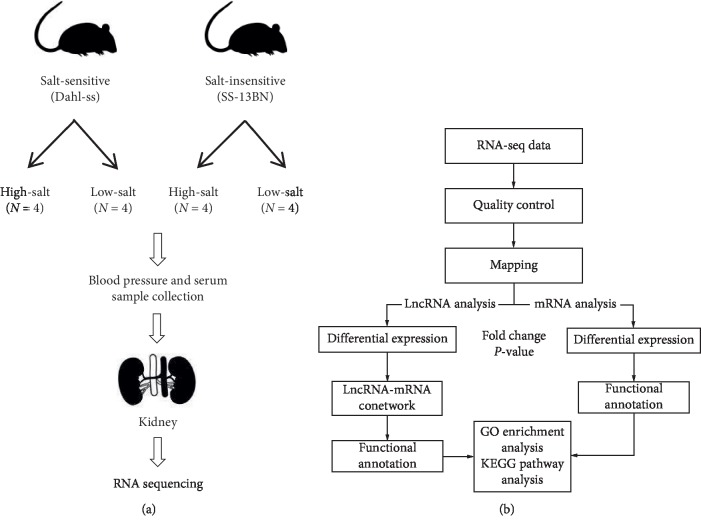
(a) Details of experimental design. (b) The schematic of RNA-seq analysis.

Sixteen specific pathogen-free rats aged 6–8 weeks, with body weights of approximately 220–280 g, were purchased from Vital River Laboratory Animal Technology (Beijing, China). Among the rats, 8 were salt-sensitive (Dahl-ss), and 8 were salt-insensitive (SS-13BN). High-salt (8% sodium chloride) and low-salt (0.25% sodium chloride) diets were administered to the salt-sensitive (SS) and salt-insensitive (SI) groups, with four rats within each group assigned randomly to the high- and low-salt subgroups, respectively (Figure 1(a)). This research was performed according to the National Institutes of Health guide for the care and use of Laboratory Animals (NIH Publications No. 8023, revised 1978), and the animals used in the experiment are males. The study was approved by the Animal Welfare and Ethics Group of the Department of Laboratory Animal Science, Fudan University (201811001Z).

### 2.2. Blood Pressure Measurements and Sample Collection

Analyses of baseline blood pressure and the selection of blood and urine samples were performed in both groups. Blood pressure was measured monthly, and blood and urine samples were collected once every two months. Blood pressure was measured by the tail-cuff method, and the mean tail arterial pressure was calculated from 3–5 successful pressure readings. The noninvasive rat tail arterial pressure monitor was purchased from Beijing Zhongshi DiChuang Science and Technology Development. Blood samples of 1–1.5 mL each were collected by retro-orbital bleeding. Serum was separated from samples by centrifugation at 3500 r/min for 30 minutes and subsequently stored at −80°C for measurement of serum sodium levels. Metabolic cages were used to collect 24 hour urine samples (diet and water were allowed ad libitum), which were stored at −80°C for urine sodium and creatinine level measurements. All tests were performed using a Beckman AU5800 automatic biochemistry analyzer (Beckman Coulter, Brea, CA, USA). After intraperitoneal injection of pentobarbital sodium (50 mg/kg), rats were sacrificed by exsanguination. Both kidneys of each rat were snap frozen in liquid nitrogen and stored at −80°C for subsequent sequencing analysis. Because the urine volumes collected in metabolic cages were not wholly accurate, the urine sodium/creatinine ratio was used to assess urine sodium levels in the rats.

### 2.3. RNA Extraction and Processing

Rat kidney RNA was extracted using a TissueLyser II (Qiagen, Valencia, CA, USA) kit. After the RNA quality test was passed, ribosomal RNA was removed from the samples using the eukaryote RiboMinus kit (Invitrogen, Carlsbad, CA, USA) in accordance with manufacturer's instructions and analyzed with an Agilent 2100 Bioanalyzer (Agilent Technology, Santa Clara, CA, USA).

### 2.4. cDNA Library Preparation and Sequencing

cDNA library preparation and sequencing steps included the following: RNA fragmentation, cDNA reverse transcription, cDNA repair, adapter ligation, and qPCR (Real-Time Quantitative Polymerase Chain Reaction) quality control. Samples were sequenced using the Illumina Hiseq3000 system (Illumina, San Diego, CA, USA).

### 2.5. RNA-seq Data Processing, Differential Gene Expression Analysis, and Correlation Analysis

The schematic of RNA-seq analysis is shown in Figure 1(b). Data quality was assessed using FastQC (quality control), and low-quality readings were removed using Trimmomatic. HiSat2 tool was used to align the remaining readings to the rat database, and matched data were annotated using the Rat Rnor_5.0. Analysis of correlations of the protein-coding genes with the ncRNAs and processed transcripts was performed. Data preprocessing and calculation of Pearson's correlation coefficients were performed using the “base” function and the “stat” package in the R platform (http://www.r-project.org). Gene quantification was performed using StringTie, and data analyses were conducted using the R language “stat” package (http://www.r-project.org). Differentially expressed genes (DEGs) were analyzed by the *t*-test based on the fold change of expression in reads per million after *p* values filtered genes. The constructed Venn diagram was analyzed using the “Venn Diagram” package; subsequently, data were subjected to principal component analysis and cluster analysis (Heat Map).

### 2.6. Analysis of Target Genes and Relevant Pathways

Kyoto Encyclopedia of Genes and Genomes (KEGG) pathway was analyzed using the Database for Annotation, Visualization and Integrated Discovery (DAVID) to observe the protein enrichment pathways, target proteins, and their relationships. Fisher's *t*-test was used to examine the validity of the data. Selective analyses were performed on pathways with *p* values <0.05.

### 2.7. Coexpression Analysis

A gene coexpression network diagram is constructed based on similarities among gene expression data. Nodes in the diagram represent genes, and genes with similar expression profiles are connected to form a network. Construction of the gene coexpression network for this study was performed using Cytoscape3.6.1.

### 2.8. Statistical Analyses

SPSS 22.0 software (IBM, Chicago, IL, USA) was used for the statistical analyses. Data are expressed as means ± standard deviation. Differences between the salt-sensitive and control groups were analyzed using the independent samples bilateral *t*-test. Differences were considered statistically significant when *p* values were <0.05.

## 3. Results

### 3.1. Blood Pressure Comparisons

High- and low-salt diets were administered to the salt-sensitive (*N*=8) and salt-insensitive groups (*N*=8), with four rats within each group assigned randomly to the high- and low-salt subgroups, respectively. Blood pressure measurements were performed in all groups at 0, 1, and 2 months and compared. The mean blood pressure value of the high salt-sensitive (SS-High) group was significantly higher (*p* < 0.05) than that of the high salt-insensitive (SI-High) group at 1 month (systolic) and 2 months (systolic and diastolic). Within the salt-sensitive group, differences in systolic blood pressure at 1 and 2 months and diastolic blood pressure at 2 months between the SS-High and SS-Low subgroups were statistically significant (*p* < 0.05) (Table 1).

### 3.2. Comparison of Serum Sodium Concentrations and Urine Sodium/Creatinine Ratios

The urine sodium/creatinine ratios of the high-salt group and low-salt group at 0 and 2 months were compared. The mean value of urine sodium/creatinine ratio of the SS-High group at 0 and 2 months was significantly higher than that of the SS-Low group (*p* < 0.05). And the mean value of urine sodium/creatinine ratio of the SI-High group at 0 and 2 months was significantly higher than that of the SI-Low group (*p* < 0.05). Within the salt-sensitive group, the mean values of the urine sodium/creatinine ratios of the SS-High group at 0 and 2 months were significantly higher than those of the SS-Low group (*p* < 0.05). Serum sodium levels of the four subgroups of rats were compared at different time points. No statistical differences were observed among these groups (Table 2).

### 3.3. Differential Expression Analysis of mRNA

**Figure 2:**
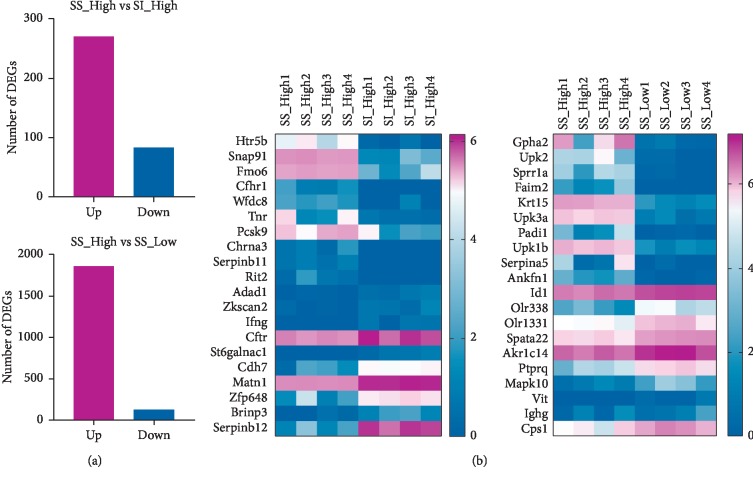
(a) Number of DEGs identified in each groups (*N*=4). (b) The heat map shows significant differences in genes in each group (*N*=4).

The mRNA analysis showed that, under high-salt intervention, there were 354 DEGs in the sensitive (SS-High) and the insensitive groups (SI-High) (*p* < 0.05). And 271 genes were upregulated, whereas 83 were downregulated. Totally, 1989 genes were differently expressed between the high-salt group (SS-High) and low-salt group (SS-Low) of salt-sensitive rats in the kidney (Figure 2(a)). 1864 genes were upregulated, and 125 genes were downregulated. The heat map also shows significant differences in genes between the two groups (Figure 2(b)).

### 3.4. Functional Enrichment Analysis

**Figure 3:**
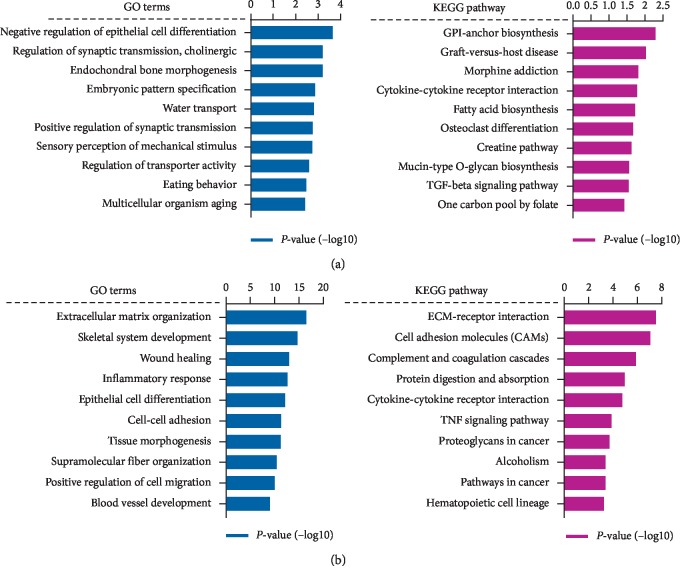
(a) The GO enrichment and KEGG pathway analysis of DEGs between SS-High group and SI-High group. (b) The GO enrichment and KEGG pathway analysis of DEGs between SS-High group and SS-Low group.

The DEGs of each comparison group were used for GO enrichment and KEGG pathway analysis. The top 10 most significantly enriched pathways of DEGs are shown in Figure 3. For high-salt intervention, the functional terms, including epithelial cell differentiation and TGF-beta signaling pathway, were enriched with DEGs between SS-High group and SI-High group. To further demonstrate the functional influence of hypertension, we analyzed the DEGs between the SS-High group and the SS-Low group. The GO enrichment and KEGG pathway analysis showed that gene related to ECM-receptor interaction, cell adhesion molecules (CAMs), inflammatory response, and blood vessel development.

### 3.5. Correlation Analysis and Coexpression Analysis between lncRNA and mRNA

**Figure 4:**
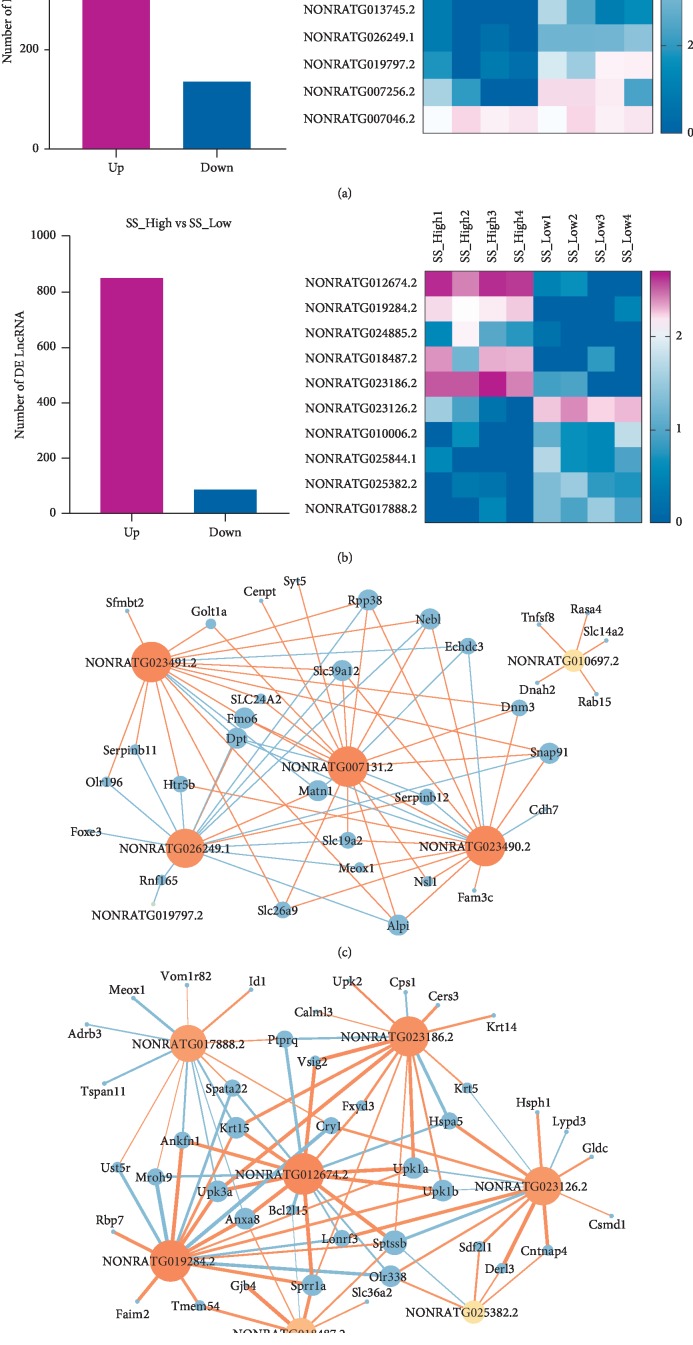
(a) Number of DE lncRNAs and heat map of top 10 significant differences genes between SS-High group (*N*=4) and SI-High group (*N*=4). (b) Number of DE lncRNAs and heat map of top 10 significant differences genes between SS-High group (*N*=4) and SS-Low group (*N*=4). (c) Coexpression analysis of lncRNA and mRNA between SS-High group (*N*=4) and SI-High group (*N*=4). (d) Coexpression analysis of lncRNA and mRNA between SS-High group (*N*=4) and SS-Low group (*N*=4).

The lncRNA analysis showed that 704 lncRNAs were differently expressed between the sensitive group (SS-High) and the insensitive groups (SI-High) (*p* < 0.05), and 936 lncRNAs were differently expressed between the high-salt group (SS-High) and low-salt group (SS-Low). The significant differences genes were showed in heat map (Figures 4(a) and 4(b)). Besides, we performed a coexpression network map to analyze the correlation between differentially expressed lncRNAs and mRNA (correlation is greater than 0.9 or less than −0.9). We found that blood pressure and angiogenesis-related genes, such as Matn1, Serpinb12, Slc39a12, and Snap91, were highly correlated with NONRATG007131.2. Therefore, it could be deduced that NONRATG007131.2 might affect blood pressure of salt-sensitive hypertensive rats (Figure 4(c)). Within the salt-sensitive groups, NONRATG012674.2 was upregulated in the high-salt group (SS-High). It could interact with hypertensive-related genes, such as Anxa8, Hspa5, and Krt15 (Figure 4(d)).

## 4. Discussion

Laboratory rats have been used to model human renal and cardiovascular disease for decades. In this study, Dahl-ss and SS-13BN rats (commonly used in salt-sensitive hypertension) were used to observe changes in blood pressure in response to diets containing different salt concentrations. Previous studies have reported several noncoding RNAs related to salt-sensitive hypertension [18–21]; however, we still have some innovations in the present study. This is the first study to analyze the difference in lncRNA and mRNA between salt-sensitive and salt-insensitive rats with different salt diets by high-throughput sequencing.

The results showed that the mean blood pressure of the SS-High group was significantly higher than that of the SI-High group at 1 and 2 months (*p* < 0.05). Within the salt-sensitive group, the mean blood pressure (systolic and diastolic) of rats fed with a high-salt diet was significantly higher at 1 and 2 months compared with rats fed with a low-salt diet (*p* < 0.05). These indicated salt sensitivity and salt concentration were two key factors for the induction of hypertension.

The mean value of urine sodium/creatinine ratio of the high-salt subgroup at 0 and 2 months was significantly higher than that of the low-salt subgroup in both sensitive group and insensitive group (*p* < 0.05). These results show that the levels of sodium intake are consistent with the levels of sodium excretion. Within the salt-sensitive group, the SS-High group had higher sodium excretion levels compared to the SS-Low group, suggesting that salt-induced hypertension in salt-sensitive rats was not caused by sodium retention in the body or reduced sodium excretion. This phenomenon indicates that there are mechanisms other than sodium that can cause hypertension in salt-sensitive hypertensive rats. Serum sodium levels of the four subgroups of rats were compared at different time points. No statistical differences were observed among these groups, and this result indicates that serum sodium is not related to salt sensitivity and salt intake.

RNA-seq analysis showed that NONRATG007131.2 was the most different LncRNA between the SS-High and the SI-High group. Through analysis of correlations between mRNAs and lncRNAs, we found that multiple RNAs were strongly correlated with differentially expressed lncRNAs, such as Matn1, Slc39a12, Serpinb12, Fmo6, Rpp38, and Snap91. These genes may regulate blood pressure changes through pathways such as blood vessel development, epithelial cell differentiation, and TGF-beta signaling pathway. Matn1 is the member of the family of matrix-derived inhibitors of neovascularization, which was found to suppress new capillary growth [22–24]. Serpinb12 may play a vital role in barrier function by providing protection of epithelial cells including matrix remodeling, fibrinolysis, and thrombosis process [25–27]. It is an extremely promising candidate gene associated with salt-sensitive hypertension. Within the salt-sensitive groups, NONRATG012674.2 was upregulated in the high-salt group (SS-High). It could interact with hypertensive-related genes, such as Anxa8, Hspa5, and Krt15 et al. Anxa8 may function as an anticoagulant that indirectly inhibits the thromboplastin-specific complex [28]. Hspa5 is a member of the heat shock protein 70 (HSP70) family, which is correlated with inflammatory markers of essential hypertension [29, 30].

The primary purpose of this study was to provide guidance and assistance for hypertension treatments for different salt-sensitive populations through observational analysis and preliminary validations of DEGs in salt-sensitive rats. Although the expression of rat and human genes is not entirely consistent, kidney samples from humans with salt-sensitive hypertension are not easily obtainable. Therefore, animal models have been used for preliminary investigations. Nonetheless, further validation of cellular functions can be conducted to prove whether they are the target genes in salt-sensitive hypertension-induced renal injury.

The field of studies of hypertension (including genetics and epigenetics) begun to focus on genetic variants that exist outside of protein-coding genes as potential drivers. The present study is the latest and most detailed research to characterize lncRNA in rat models of hypertension and kidney disease. The data presented in this study should lay the foundation for investigating ncRNA as a candidate for regulating the cause of hypertension.

## Figures and Tables

**Table 1:** Comparison of mean blood pressure in rats ($\bar{x}\pm s$) (mmHg).

		SS-High (*N*=4)	SS-Low (*N*=4)	SI-High (*N*=4)	SI-Low (*N*=4)
0 months	SBP	128 ± 2.94	123.75 ± 4.79	126.5 ± 1.2	123.25 ± 5.68
	DBP	86.5 ± 2.38	81 ± 1.82	88.26 ± 1.26	80 ± 2.16

1 month	SBP	164.5 ± 10.34^*∗*#^	135.25 ± 4.43	133.25 ± 2.22	128.75 ± 2.5
	DBP	103.75 ± 5.85	88.5 ± 5.45	90 ± 4.76	83.25 ± 3.59

2 months	SBP	196.75 ± 8.38^*∗*#^	139.75 ± 2.21	139.5 ± 4.80	133 ± 2.83
	DBP	144.25 ± 4.35^*∗*#^	88.75 ± 2.75	91.5 ± 2.38	85.25 ± 4.57

SBP: systolic blood pressure; DBP: diastolic blood pressure. ^*∗*^SS-High vs. SI-High, *p* < 0.05; ^#^SS-High vs. SS-Low, *p* < 0.05.

**Table 2:** Comparison of mean values of serum sodium and urinary sodium/urine creatinine in rats ($\bar{x}\pm s$) (mmHg).

	SS-High (*N*=4)	SS-Low (*N*=4)	SI-High (*N*=4)	SI-Low (*N*=4)
Serum sodium (mmol/l)				
0 months	135.88 ± 9.49	135.03 ± 7.75	134.4 ± 3.51	135.48 ± 3.78
2 months	140.7 ± 1.76	141.38 ± 2.43	141.1 ± 1.78	138.23 ± 1.30

Sodium/urine creatinine				
0 months	12.17 ± 1.63^*∗*#^	4.91 ± 0.28	6.13 ± 0.27^*∗*^	4.19 ± 0.22
2 months	202.37 ± 64.9^*∗*#^	10.61 ± 1.12	155.91 ± 13.32^*∗*^	6.85 ± 0.34

^*∗*^Sensitive group: SS-High vs. SS-Low, *p* < 0.05; insensitive group: SI-High vs. SI-Low, *p* < 0.05. ^#^SS-High vs. SI-High, *p* < 0.05.

## Data Availability

The data sets used and/or analyzed during the current study are available from the corresponding author on reasonable request.
